# Glial cells diverge in fly brain evolution

**DOI:** 10.1371/journal.pbio.3003136

**Published:** 2025-04-30

**Authors:** Yaoyu Jiao, Trevor R. Sorrells

**Affiliations:** 1 Department of Genetics, Yale School of Medicine, New Haven, Connecticut, United States of America; 2 Howard Hughes Medical Institute, New Haven, Connecticut, United States of America; 3 Wu Tsai Institute, Yale University, New Haven, Connecticut, United States of America

## Abstract

How animal brains evolve to support ecological specialization is poorly understood. This Primer explores a recent PLOS Biology study which reveals that glial cells show the most dramatic molecular and cellular changes in the brains of fruit flies adapted to a toxic niche, highlighting their underappreciated role in brain evolution.

## Teaser

How animal brains evolve to support ecological specialization is poorly understood. A recent PLOS Biology study reveals that glial cells show the most dramatic molecular and cellular changes in the brains of fruit flies adapted to a toxic niche, highlighting their underappreciated role in brain evolution.

At nature’s grand buffet, drosophilid flies have made strikingly different dining choices. Generalists such as *Drosophila melanogaster* and *Drosophila simulans* sample widely from the menu of decaying fruits and vegetation. In contrast, a closely related species, *Drosophila sechellia*, is a specialist that feeds exclusively on the noni fruit *Morinda citrifolia*, which is toxic to other drosophilids. Previous studies have primarily focused on olfactory receptors and neurons enabling the host specialization in *D. sechellia*’s peripheral nervous system [1–3]. Yet, how the brain evolves to accommodate this ecological shift remained unexplored until a new PLOS Biology study from Lee and colleagues [4].

**Fig 1:**
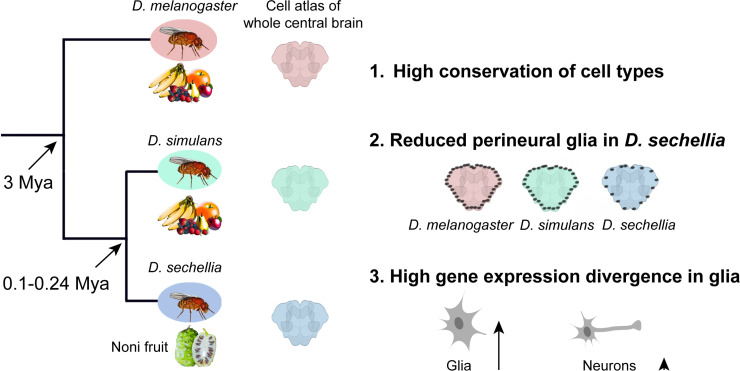
Comparative atlases of three closely related *Drosophila* reveal a glial role in fly brain evolution. Phylogenetic relationships and approximate divergence times are indicated at left. The authors generated single-nucleus RNA sequencing of the whole central brains of two generalists, *Drosophila melanogaster* and *Drosophila simulans*, and a specialist on noni fruit, *Drosophila sechellia*. This allowed them to estimate the divergence in gene expression and abundance in brain cell types, revealing that glia diverge more quickly than neurons.

Investigation of brain evolution has traditionally relied on comparing brain structures and behaviors across species, attempting to link anatomical differences to ecological adaptations [5]. Recent advances in single-cell transcriptomics offer a powerful new toolkit for studying brain evolution at the levels of gene expression and cell type. Leveraging the phylogenetic distance of *D. melanogaster*, *D simulans*, and *D. sechellia* and their different ecological niches, the authors generated 1× coverage single-cell transcriptomic atlases of central brains (~50,000 cells per species) using single-nucleus RNA sequencing [4]. This comparative analysis, the first in whole central brains of any animal at single-cell resolution (see Fig 1), reveals how gene expression evolves at the cell type level and advances these species as a model for behavioral evolution.

The authors found that the brain is highly conserved across these three fruit fly species with most cell types exhibiting similar frequencies and gene expression patterns [4]. However, when compared to *D. melanogaster*, the specialist *D. sechellia* showed greater molecular and cellular divergence than their closely related generalist counterpart *D. simulans* [4]. This divergence may be due to the dramatic ecological shift of *D. sechellia*.

A long-standing debate in the field of evolutionary biology is to what degree differences in species can be ascribed to adaptation or non-adaptive processes such as mutation and drift. *D. sechellia* has a much smaller population size than *D. simulans*, decreasing the power of selection relative to genetic drift. This study system provides an excellent opportunity to understand the relative contributions of adaptive and non-adaptive changes to brain evolution between species.

The authors’ most unexpected discovery concerns glia. Meaning ‘glue’ in ancient Greek, glia were long considered to be exclusively supporters of the nervous system, responsible for structural scaffolding, nutrient supply, and waste removal. Neurons have generally received more attention for their role in information processing and behavior control. Lee and colleagues revealed that glial cells—particularly perineurial glia forming the blood-brain barrier—displayed significant reductions in cell abundance and extensive gene expression changes in *D. sechellia* [4]. These results were rigorously validated through fluorescence *in situ* hybridization. The gene expression changes in glia were enriched for metabolic pathways, consistent with noni fruit’s unique nutritional profile (low sugars, high toxins). These findings provide intriguing evidence that nervous system adaptation may occur through glial cells in *D. sechellia*’s ecological specialization to noni fruit.

In recent years, glia have been shown to play many critical roles in central nervous system function [6]. Several recent studies using single-cell sequencing techniques have uncovered surprising roles of glia in regulating insect behavior. For example, *Camponotus floridanus* ants have two developmental castes, majors that defend the nest and minors that forage for food. Comparative single-cell RNA sequencing of brains identified juvenile hormone-degrading enzymes in perineurial glia that form the blood-brain barrier as key regulators of these caste-specific behaviors [7]. A study of *D. melanogaster* brains from different sleep and wakefulness states revealed that glial cells integrate homeostatic and circadian processes [8]. Furthermore, blood-feeding causes *Aedes aegypti* mosquitoes to suppress their appetite during which time there are extensive transcriptional changes in glia but not neurons [9]. Lee and colleagues add a new piece: glia’s rapid evolution in *D. sechellia* highlights their role as evolutionary innovators in the brain. Why might glia be more evolutionarily labile? Unlike neurons, which face stringent constraints to maintain conserved synaptic and circuit architectures, glia’s metabolic and homeostatic roles may permit greater flexibility during niche adaptation.

This study is a landmark for evolutionary neurobiology, expanding on comparative studies of specific brain regions to the whole central brain. This has allowed the comparison of evolutionary rates between cell types, with the finding that glial cells evolve much more rapidly than neurons. Like other landmark studies, this raises numerous additional questions. Is rapid glial evolution found in other systems? Do these changes result in adaptive differences between species or do they reflect lower purifying selection on glial cells? The three closely related *Drosophila* species used by Lee and colleagues offer a powerful model for investigating brain evolution. Decades of neurobiology research have generated a wealth of research tools in *D. melanogaster* that are beginning to be deployed in closely related species. The integration of neurobiology, molecular and genetic techniques, and population genomics will provide new insight into the evolution of the brain and behavior.

## References

[1] Prieto-Godino LL, Rytz R, Cruchet S, Bargeton B, Abuin L, Silbering AF, et al. Evolution of acid-sensing olfactory circuits in drosophilids. Neuron. 2017;93(3):661–676.e6. doi: 10.1016/j.neuron.2016.12.024 28111079

[2] Auer TO, Khallaf MA, Silbering AF, Zappia G, Ellis K, Álvarez-Ocaña R, et al. Olfactory receptor and circuit evolution promote host specialization. Nature. 2020;579(7799):402–8. doi: 10.1038/s41586-020-2073-7 32132713 PMC7100913

[3] Álvarez-Ocaña R, Shahandeh MP, Ray V, Auer TO, Gompel N, Benton R. Odor-regulated oviposition behavior in an ecological specialist. Nat Commun. 2023;14(1):3041. doi: 10.1038/s41467-023-38722-z 37236992 PMC10219952

[4] Lee D, Shahandeh M, Abuin L, Benton R. Comparative single-cell transcriptomic atlases of drosophilid brains suggest glial evolution during ecological adaptation. PLoS Biology. 2025;23(4):e3003120. doi: 10.1371/journal.pbio.3003120 40299832 PMC12040179

[5] Roberts RJV, Pop S, Prieto-Godino LL. Evolution of central neural circuits: state of the art and perspectives. Nat Rev Neurosci. 2022;23(12):725–43. doi: 10.1038/s41583-022-00644-y 36289403

[6] Allen NJ, Lyons DA. Glia as architects of central nervous system formation and function. Science. 2018;362(6411):181–5. doi: 10.1126/science.aat0473 30309945 PMC6292669

[7] Ju L, Glastad KM, Sheng L, Gospocic J, Kingwell CJ, Davidson SM, et al. Hormonal gatekeeping via the blood-brain barrier governs caste-specific behavior in ants. Cell. 2023;186(20):4289–4309.e23. doi: 10.1016/j.cell.2023.08.002 37683635 PMC10807403

[8] Dopp J, Ortega A, Davie K, Poovathingal S, Baz E-S, Liu S. Single-cell transcriptomics reveals that glial cells integrate homeostatic and circadian processes to drive sleep-wake cycles. Nat Neurosci. 2024;27(2):359–72. doi: 10.1038/s41593-023-01549-4 38263460 PMC10849968

[9] Goldman O, DeFoe A, Qi Y, Jiao Y, Weng S-C, Houri-Zeevi L, et al. Mosquito Cell Atlas: a single-nucleus transcriptomic atlas of the adult *Aedes aegypti* mosquito. bioRxiv. 2025. doi: 10.1101/2025.02.25.639765 PMC12767863 41172998

